# High-level expression and characterization of a chimeric lipase from *Rhizopus oryzae* for biodiesel production

**DOI:** 10.1186/1754-6834-6-29

**Published:** 2013-02-21

**Authors:** Xiao-Wei Yu, Chong Sha, Yong-Liang Guo, Rong Xiao, Yan Xu

**Affiliations:** 1 State Key Laboratory of Food Science and Technology, the Key Laboratory of Industrial Biotechnology, Ministry of Education, School of Biotechnology, Jiangnan University, 1800 Lihu Avenue, Wuxi 214122, Jiangsu, China; 2 Center for Advanced Biotechnology and Medicine, Department of Molecular Biology and Biochemistry, Rutgers University, Piscataway, NJ 08854, USA

**Keywords:** Biodiesel, Lipase, Chimera, Prosequence, *Rhizopus oryzae*, *Pichia pastoris*, Tung oil

## Abstract

**Background:**

Production of biodiesel from non-edible oils is receiving increasing attention. Tung oil, called “China wood oil” is one kind of promising non-edible biodiesel oil in China. To our knowledge, tung oil has not been used to produce biodiesel by enzymatic method. The enzymatic production of biodiesel has been investigated extensively by using *Rhizopus oryzae* lipase as catalyst. However, the high cost of *R. oryzae* lipase remains a barrier for its industrial applications. Through different heterologous expression strategies and fermentation techniques, the highest expression level of the lipase from *R. oryzae* reached 1334 U/mL in *Pichia pastoris*, which is still not optimistic for industry applications.

**Results:**

The prosequence of lipases from *Rhizopus* sp. is very important for the folding and secretion of an active lipase. A chimeric lipase from *R. oryzae* was constructed by replacing the prosequence with that from the *R. chinensis* lipase and expressed in *P. pastoris*. The maximum activity of the chimera reached 4050 U/mL, which was 11 fold higher than that of the parent. The properties of the chimera were studied. The immobilized chimera was used successfully for biodiesel production from tung oil, which achieved higher FAME yield compared with the free chimeric lipase, non-chimeric lipase and mature lipase. By response surface methodology, three variables, water content, methanol to tung oil molar ratio and enzyme dosage were proved to be crucial parameters for biosynthesis of FAME and the FAME yield reached 91.9±2.5% at the optimized conditions by adding 5.66 wt.% of the initial water based on oil weight, 3.88 of methanol to tung oil molar ratio and 13.24 wt.% of enzyme concentration based on oil weight at 40°C.

**Conclusions:**

This is the first report on improving the expression level of the lipase from *R. oryzae* by replacing prosequences. The immobilized chimera was used successfully for biodiesel production from tung oil. Using tung oil as non-edible raw material and a chimeric lipase from *R. oryzae* as an economic catalyst make this study a promising one for biodiesel applications.

## Background

During the past decades biodiesel (monoalkyl esters of long-chain fatty acids) is receiving increasing attention as an alternative diesel fuel because of its favorable properties, environmental benefits and the fact that is derived from the renewable biological resources [1,2]. At present, biodiesel is mainly produced from edible oils (more than 95%), such as soybean oil, rapeseed oil and palm oil, which leads to global imbalance to the food supply and demand market. One alternative way is to produce biodiesel from low cost non-edible oils. Most of the non-edible plants can be grown in wasteland and infertile land, which allows the use of wasteland to produce oil crops for biodiesel production without the need to compete with food crops for the limited arable land [3,4]. Thus, focus should be shifted to the sustainable non-edible resources which will be crucial determinants in the popularization of biodiesel.

Tung oil, called “China wood oil” is one kind of promising non-edible biodiesel oil in China. Tung trees are spread widely in China. The oil content of the seeds and whole nuts is approximately 21 and 41 wt.%, respectively. The productivity of tung oil is from 300 to 450 kg/ha, obtained by pressing the seeds of the tung tree. Unlike vegetable oils that contain high amounts of saturated fatty acids, tung oil is composed of more than 60% unsaturated fatty acid, mainly 9Z,11E,13E-α-elaeostearic acid [5,6]. Generally, saturated fatty acid methyl esters have good oxidation stability and poor low temperature properties. On the contrary, unsaturated fatty acid methyl esters have good low temperature properties and poor oxidation stability [6]. The shortcomings of tung oil methyl esters could be solved by blending with palm, coconut and canola oil biodiesels [5,7].

Biodiesel could be produced by chemical or enzymatic methods according to the catalysts employed in the process. Contrary to chemical catalysts, enzymatic method does not form soaps and can esterify both FFA and TAG in one step without the need of a subsequent washing step [2,8]. This is especially the case when using feeds high in FFA such as tung oil. To our knowledge, tung oil has not been used to produce biodiesel by enzymatic method, except for several reports by chemical method [6,7,9,10].

Nowadays biodiesel production by lipase-catalyzed transesterification becomes an interesting prospect in an industrial scale. The enzymatic production of biodiesel has been investigated extensively by using *Rhizopus oryzae* lipase (ROL) [11,12]. The free *R. oryzae* lipase F-APl5 (Amano) catalyzed the methanolysis of soybean oil which reached 80 wt.% yield of fatty acid methyl esters (FAME) by stepwise additions of methanol to the reaction mixture in the presence of 4~30 wt.% water [13]. The crude recombinant *R. oryzae* lipase LY6 by *Pichia pastoris* immobilized on anion exchange resin Amberlite IRA-93 was used to biosynthesis biodiesel from soybean oil and the highest biodiesel yield was achieved at 90.5% [14].

However, the high cost of the catalyst ROL remains a barrier for its industrial applications. In order to bring the cost down, one of the options is to enhance the expression level of *R. oryzae* lipases. The production of active *R. oryzae* lipases has been performed in *Escherichia coli*[15], in *Saccharomyces cerevisiae*[16,17] and in *P. pastoris*[18-20]. Lorenzo *et al.*[15] successfully expressed the *R. oryzae* prolipase (proROL) in a soluble form in *E. coli* with an activity of 166 U/mL (protein concentration 1.5 mg/mL). Takahashi *et al.*[17] reported that the activity of proROL by *S. cerevisiae* reached 2.88 U/mL (protein concentration 28.0 mg/L, *OD*_600_ about 90) after 120 h of cultivation in YPD medium. The activity of *R. arrhizus* prolipase expressed in *P. pastoris* was obtained at 140 U/mL (4 375 U/g dry cell weight and 91 mg enzyme/L broth) after 92 h of cultivation in complex medium [21]. The activity of the mature lipase from *R. oryzae* (mROL) expressed in *P. pastoris* reached 500 U/mL (60 g wet cell weight/L and 60 mg enzyme/L) [20]. And the activity of this lipase was further improved to 1334 U/mL (about 48 g dry cell weight/L) by a methanol feeding strategy [18], which was the highest expression level ever reported. However, through different heterologous expression strategies and fermentation techniques, the expression level of *R. oryzae* lipases is still not optimistic for industry applications.

The prosequenes in some proteolytic enzyme precursors inhibit the activity of the mature portions, while some of the prosequences have the function to help folding of the mature portions, such as subtilisin E of *Bacillus subtilis*[22], carboxypeptidase Y of *S. cerevisiae*[23], and lipases from *Rhizopus* sp. [24,25]. The lipase secreted from *R. oryzae*, similar to the lipases from *R. chinensis*, *Rhizomucor miehei* and *Fusarium heterosporum*, is synthesized as a precursor form with a presequence (23 amino acid residues) and a prosequence (97 amino acid residues) at the N-terminal side of the mature portion (268 amino acid residues) [24,25]. In *E. coli*, the activity of the prolipase could reach 100 U/mL, while the mature portion of ROL was expressed as an insoluble form without activity. The mutation studies demonstrated that the prosequence of ROL seems to facilitate the folding by providing an intramolecular thiol-disulfide reagent, and proROL is also significantly more stable against thermal inactivation than mROL [25,26]. For the expression of *R. arrhizus* lipases in *P. pastoris*, the pro-form lipase (r28RAL) and the mature portion of the lipase in the supernatant reached 91 mg/L and 80 mg/L, respectively [21]. Takahashi *et al*. [24] explored the role of the prosequence of ROL expressed in *S. cerevisiae* and indicated that the prosequence might support the correct folding of its mature portion and its subsequent secretion from the yeast cells.

In this study, we constructed a chimeric lipase from *R. oryzae* by replacing the prosequence with that from *R. chinensis* lipase and successfully expressed in *P. pastoris* at high-level. The chimera was characterized and its performance for biodiesel production from non-edible tung oil was investigated by response surface methodology.

## Results and discussion

### Design of the chimeric lipase

*Rhizopus* sp. are mainly divided into three groups, including *R. microsporus, R. oryzae, R. stolonifer*[27]*.* In our previous study, a lipase gene (GenBank accession No. EF405962) cloned from *R. chinensis* CCTCC M201021 (belongs to *R. microsporus*) was expressed at high-level (2130 U/mL) in *P. pastoris* which was about 580 times higher than that of the wild-type *R. chinensis* lipase [28,29]. We noticed a big gap between the expression level of the *R. chinensis* lipase (RCL) and the *R. oryzae* lipase. *Rhizopus* sp. lipases are synthesized as prepro-proteins. *In vivo* expression and *in vitro* refolding experiments of *R. oryzae* lipase showed that the prosequence was very important for the folding and secretion of an active lipase [24-26]. Through analysis, the deviation of the prosequences between RCL and ROL let us come up with an idea to construct a chimeric lipase from *R. oryzae* by replacing the prosequence with that from *R. chinensis* lipase in order to improve the expression level of the lipase from *R. oryzae*.

**Figure 1:**
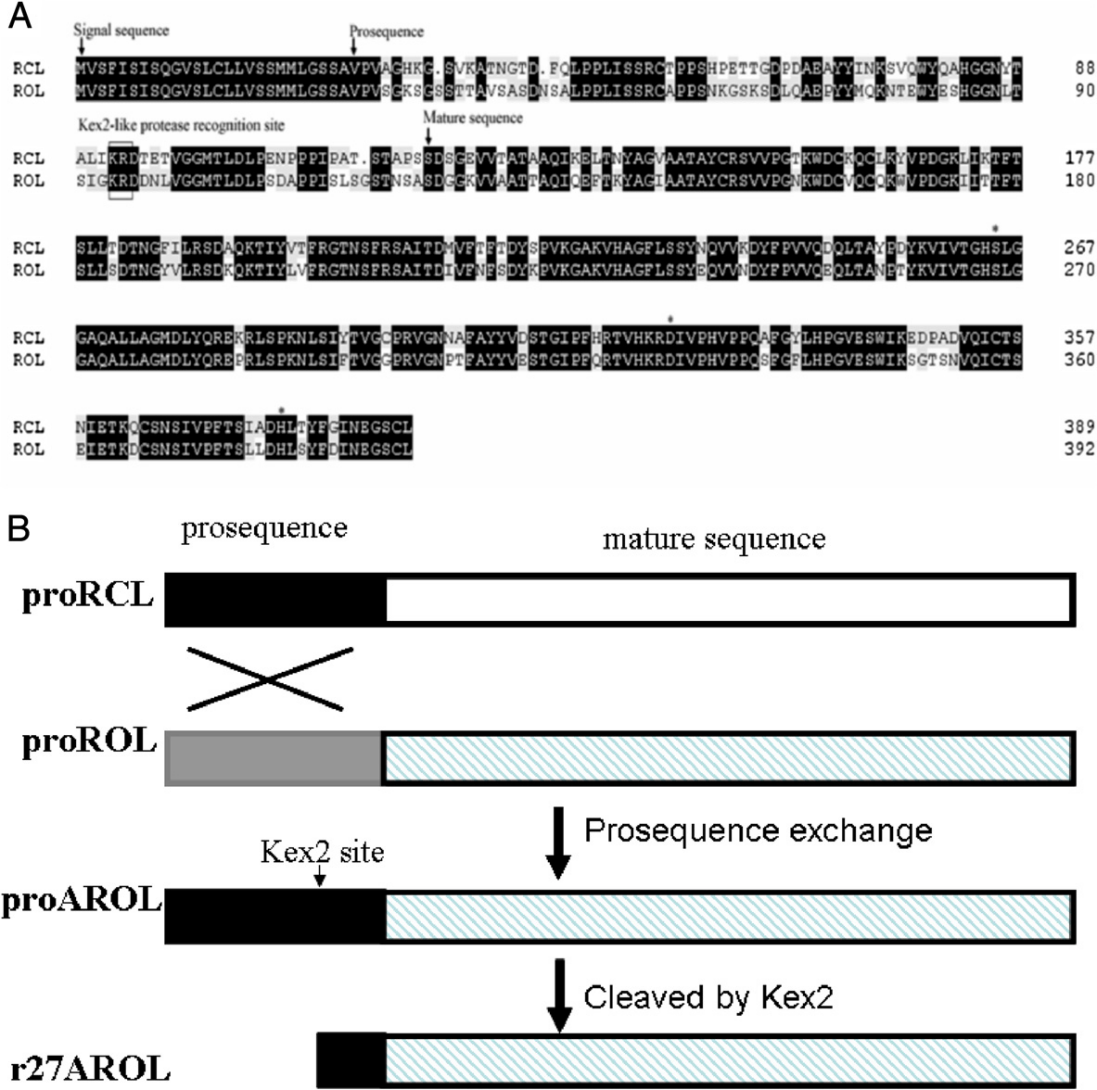
**(A) Alignment of amino acid sequences from *R. oryzae *** **lipase and *R. chinensis *** **lipase; (B) Scheme of the chimeric lipases proAROL and r27AROL.**

The cloned DNA sequence of the prepro-lipase gene from *R. oryzae* XY1 was 1176 bp shared 99.75% with the *R. oryzae* lipase gene sequence (GenBank accession No. AF229435) and 100% homology with the amino acid sequence. Amino acid sequence alignment shows that *R. oryzae* lipase was most closely related to RCL with 75.6% homology (Figure 1A). They both contain the conserved catalytic triad S-H-D indicated by star (Figure 1A). However, the prosequence from the *R. oryzae* lipase only shared 47.4% identity with that from the *R. chinensis* lipase. Compared to the *R. oryzae* lipase, the much higher expression level of the *R. chinensis* lipase in *P. pastoris* was probably contributed by the different amino acid composition of the prosequence. The chimeric *R. oryzae* lipase was constructed by replacing the prosequence with that from the *R. chinensis* lipase, named as proAROL (Figure 1B), in an aim to improve the expression level of the lipase from *R. oryzae*.

### Expression and properties of the chimeric lipase

**Figure 2:**
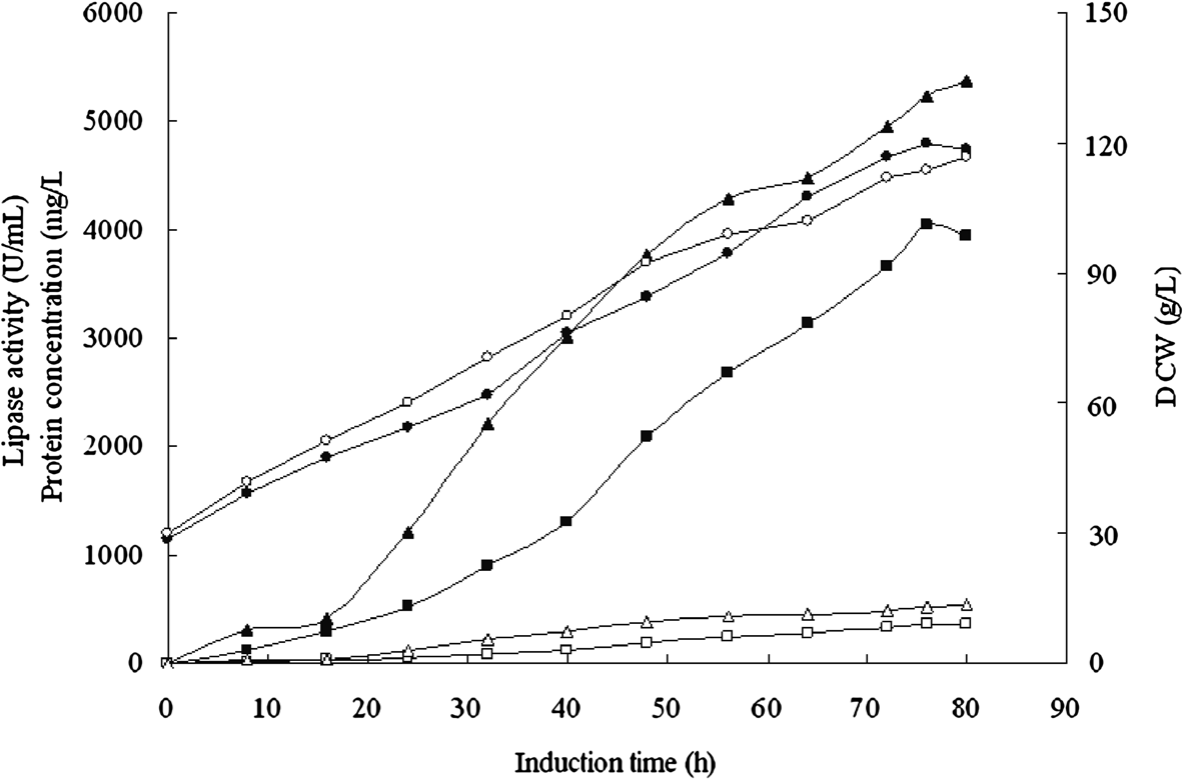
**Lipase activity, protein concentration, cell concentration profiles of GS115/pPIC9K-proROL and GS115/pPIC9K-proAROL.** Lipase activity by *p*NPP assay: (▪) chimera, (□) parent; Protein concentration: (▴) chimera, (Δ) parent; Cell concentration: (•) chimera, (○) parent.

**Figure 3:**
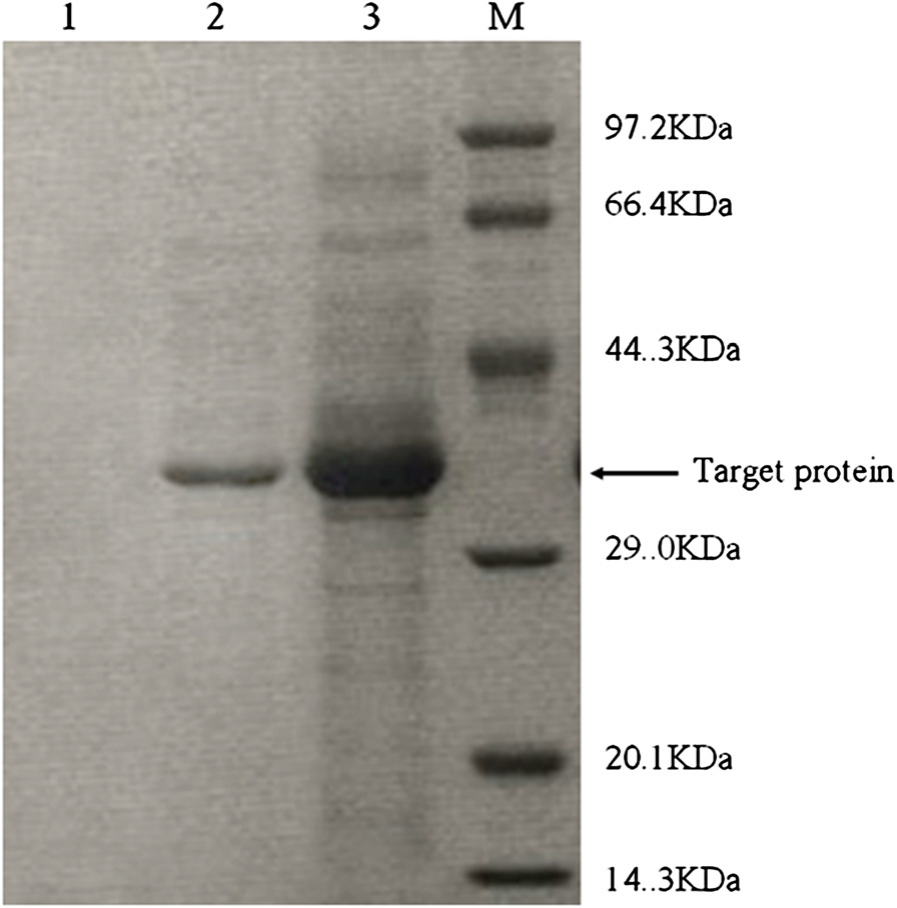
**SDS-PAGE analysis of recombinant lipases.** Lane 1: supernatant of GS115/pPIC9K as negative control; Lane 2: supernatant of GS115/pPIC9K-proROL; Lane 3: supernatant of GS115/pPIC9K-proAROL.

As shown in Figure 2, after replacing the prosequence, the activity of the chimeric lipase proAROL reached the maximum of 4050 U/mL by *p*NPP assay after induction of 76 h, which was 11 fold higher than that of the parent. It was probably that the increased lipase activity of the chimera was induced by an increased secretion or an increased catalytic rate. To explore the reason, we investigated the differences of the total protein concentrations in the supernatant, cell concentrations and the kinetic parameters between the chimeric lipase and the non-chimeric lipase. The cell concentrations (DCW, dry cell weight) were very similar, while the total protein concentration of the supernatant expressed by GS115/pPIC9K-proAROL increased significantly by 9.6 times, which was also evident in SDS-PAGE (Figure 3). The specific lipase production (*p*NPP assay) reached 33 750 U/g dry cell weight for the chimeric lipase and 3 068 U/g dry cell weight for the non-chimeric lipase, and the specific lipase activity reached 779 U/mg protein by *p*NPP assay (2 493 U/ mg protein by pH-stat assay) for the chimeric lipase and 681 U/mg protein by *p*NPP assay (2 078 U/ mg protein by pH-stat assay) for the non-chimeric lipase. On SDS-PAGE the target band of the chimera was much thicker than that of the parent and both recombinant lipases migrated as a single band with a molecular mass of about 37 kDa, which was lower than the calculated molecular weights of proROL and proAROL (39 kDa). We further purified the chimera and non-chimera for analysis of the N-terminal amino acid sequences and enzymatic properties. The N-terminal sequence of the secreted chimeric lipase, named as r27AROL, was D-T-E-T-V-G-G, corresponding to 27 amino acids of C-terminal part of the prosequence of proAROL, and the N-terminal sequence of the secreted wild type, named as r28ROL, was D-D-N-L-V-G-G, which retained 28 amino acids of C-terminal part of the prosequence of proROL. The results suggested that the secreted lipases was cleaved by Kex2-like protease in *P. pastoris* at the recognition site Lys-Arg of the prosequences, which was also observed in other reports [28,30]. The kinetic parameters were determined using *p-*nitrophenyl palmitate (*p*NPP) as substrate. The interfacial kinetic parameter *K*_m_^*^ value for the chimera was not changed compared to the parent and the *k*_cat_^*^ value was 1.32 times higher than that of the parent. The results suggested that the increased secretion of the chimeric lipase was the main factor for the much higher lipase activity of the chimera in the supernatant but the improved catalytic rate of the chimera only contributed a little. The highest expression level previously reported on a mature *R. oryzae* lipase expressed in *P. pastoris* was 1334 U/mL (pH-stat assay using olive oil emulsion as substrate) with the specific lipase production of 27 791 U/g dry cell weight [18], which was lower than the expression level of the chimera in this study. However, these values are not fully comparable since different activity assays with different substrates and conditions have been used. In order to reach a more comparable level, the activity of the same chimeric lipase sample was measured in parallel by both *p*NPP assay and pH-stat assay. The results indicated that the lipolytic activity values reported in the present work were an underestimation since the activity by pH-stat assay (12 960 U/mL) was about 3 times higher than that by *p*NPP assay (4050 U/mL).

Both lipases exhibited maximum activity at pH 8.5 and could keep more than 80±2% of the maximum activity after incubating at pH 7.0~9.0 for 24 h. However, the lipase activity decreased dramatically when temperature was over 50°C or pH was over 10.0. The optimum temperature for both lipases was 40°C. r27AROL could keep 85±3% of the maximum activity after incubating for 1 h at 40°C, and r28ROL retained 70±2% residual activity at the same condition. The prosequence from *Rhizopus* sp. not only supports folding of the lipases but also has the function to improve the thermostability of the prolipase compared with the mature lipase, such as the *R. oryzae* lipases expressed in *E. coli*[26], the *R. chinensis* lipases [28] and the *R. arrhizus* lipases expressed in *P. pastoris*[21]. In agreement with these reports, the thermostability of the mature *R. oryzae* lipase by *P. pastoris* in this study was also much lower than those of r28ROL and r27AROL, which retained 55±3% residual activity with the same heat treatment.

The mechanism of the prosequence of the lipases from *Rhizopus* sp. seems to be more complicated. In the case of proROL produced in *E. coli*, Beer *et al.*[26] presumed that cysteine-30 in the prosequence might play a key role in facilitating the folding of the enzyme and work as an intramolecular disulfide reagent, analogously to the role of the cysteine residue in the prosequence of pro-bovine pancreatic trypsin inhibitor [31]. While on the contrary Takahashi *et al.*[24] found that the mutation from cysteine-30 to serine in the prosequence of proROL expressed in *S. cerevisiae* did not affect the activity in either extra- or intracellular fractions. They proposed a model on the behavior of ROL in the ER lumen of *S. cerevisiae*. ROL with the wild-type prosequence is folded correctly by the function of the prosequence in the ER lumen and secreted extracellularly. Certain deletions in the prosequence block correct folding of the mature portion, so that ROLs with the mutated prosequences are retained in the ER lumen. Moreover, N-linked glycosylation might also influence the secretion and thermostability of enzymes [32,33]. The lipase from *R. oryzae* has three potential sites of N-glycosylation, one being in the prosequence and others in the mature portion [24]. The mutation of the glycosylation site in the prosequence of this enzyme did not affect either the secretion of active lipase [24] or the thermostability of proROL [30]. However, the prosequence from *R. chinensis* lipase contained three potential N-glycosylation sites, which might be the contributor for the high secretion and thermostability for the chimera r27AROL. Further analysis is necessary to make clear the differences between the prosequences from *R. oryzae* lipase and *R. chinensis* lipase. And to elucidate the structural relationship between the prosequence and mature portion, crystallographic analysis is ongoing in our further research.

### Comparison of the production of FAME from tung oil by the lipases

**Figure 4:**
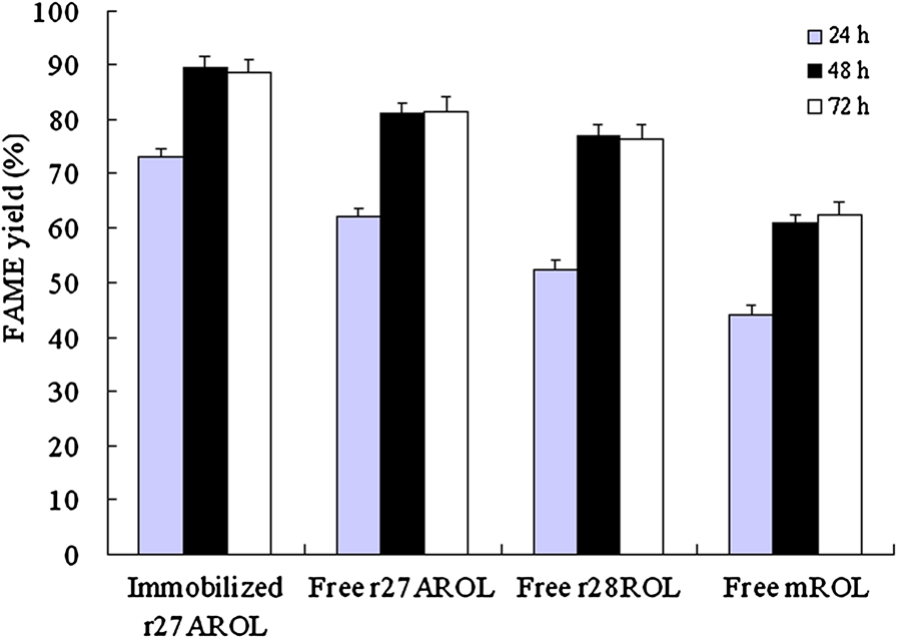
**Bioconversion of tung oil to fatty acid methyl esters by the immobilized r27AROL, free r27AROL, free r28ROL and free mROL.** The reaction was carried out by adding 81 U lipase into 10 g tung oil, methanol to oil molar ratio of 3.5:1 (Methanol was added by four steps.) at 4.5 wt.% water content in a 40°C shaker.

Immobilization is the key to the operational performance of an enzyme in industrial processes, particularly for reuse in nonaqueous media [34]. Resin has been often used for immobilization of enzymes for use in nonaqueous media, which involves dispersal of enzyme over a large surface. The chimeric lipase r27AROL was immobilized on anion exchange resin Amberlite IRA-93 and the performance of FAME production by the lipases was compared among the immobilized r27AROL and the free enzymes. Figure 4 shows the FAME formation at 24 h, 48 h and 72 h in the reaction media at 40°C. All four enzymes showed a higher conversion at 48 h than that at 24 h and to extend the reaction time to 72 h did not further increase the FAME yields. Under the same indicated reaction condition the FAME yield by immobilized r27AROL was higher than all three free lipases, which reached 89.5±2.2% at 48 h. The immobilized enzymes are generally known to give better transformation rates in nonaqueous media due to the larger surface area of the immobilized biocatalyst. On the contrary, free enzyme usually suffers mass transfer problem in low water media [34]. The free chimeric r27AROL exhibited better performance than the free non-chimeric r28ROL and free mature mROL, probably due to the different thermostability. r27AROL was the most thermostable lipase among them, while mROL without the prosequence showed the lowest thermostability.

### Optimization of the production of FAME by response surface methodology

Response surface methodology (RSM), one of the global optimization methods, helps in evaluating the effective factors and in building models to study interaction, and to select optimum conditions of variables for a desired response. In this study, RSM was adopted to optimize the process of methanolysis of tung oil to FAME by the immobilized chimeric lipase from *R. oryzae*. Based on the results obtained from single factor experiments and other most commonly used factors in the transesterification reactions catalyzed by *R. oryzae* lipases [13,35,36], the major factors affecting the transesterfication were water content, ratio of methanol to oil, and enzyme dosage. A five-level-three-factor central composite rotatable design (CCRD) was adopted to evaluate the effects of the above three factors on the FAME yield. The corresponding CCRD and experimental data were shown in Table 1 and Table 2, respectively. The data was fitted to quadratic polynomial model using the software Design-Expert 8.0.7.1, and the polynomial model for the yield of FAME was given below (in terms of actual factors):

(1) $$\begin{array}{ll} \mathrm{Fame}\;\mathrm{yield}\;\left(\%\right)= & -112.744+23.289A+24.826B\\ & +13.794C-0.700\mathrm{AB}-0.376\mathrm{AC}\\ & -0.167\mathrm{BC}-1.378A^{2}-2.405^{2}\\ & -0.416C^{2} \end{array}$$

**Table 1:** Independent variables: coded and real value in center composite rotatable design

**Variables**	**Symbols**	**Levels**				
		**−1.68 (−α)**	**−1**	**0**	**+1**	**+1.68(+α)**
Initial water added based on oil weight (wt.%)	A	0.30	2.00	4.50	7.00	8.70
Methanol to tung oil molar ratio (mol/mol)	B	0.98	2.00	3.50	5.00	6.02
Enzyme concentration based on oil weight (wt.%)	C	8.3	10.00	12.50	15.00	16.70

**Table 2:** Central composite rotatable design arrangement and responses

	**Variable levels**			**Yield (%)**	
**Design point**	**A: water content (wt. %)**	**B: Methanol to oil molar ratio**	**C: Enzyme concentration (wt. %)**	**Experimental**	**Predicted**
1	2.00	2.00	10.00	50.7	51.0
2	7.00	2.00	10.00	81.4	79.7
3	2.00	5.00	10.00	70.2	65.8
4	7.00	5.00	10.00	81.2	83.9
5	2.00	2.00	15.00	65.6	62.6
6	7.00	2.00	15.00	77.7	81.8
7	2.00	5.00	15.00	73.4	74.8
8	7.00	5.00	15.00	84.2	83.6
9	0.30	3.50	12.50	46.2	49.4
10	8.70	3.50	12.50	83.7	80.9
11	4.50	0.98	12.50	67.2	67.3
12	4.50	6.02	12.50	80.8	81.2
13	4.50	3.50	8.30	75.8	77.5
14	4.50	3.50	16.70	88.1	86.9
15	4.50	3.50	12.50	92.1	89.5
16	4.50	3.50	12.50	90.9	89.5
17	4.50	3.50	12.50	90.1	89.5
18	4.50	3.50	12.50	90.3	89.5
19	4.50	3.50	12.50	86.1	89.5
20	4.50	3.50	12.50	87.7	89.5

Where FAME yield (%) is the response value, A is the initial water added based on oil weight (wt. %), B is methanol to tung oil molar ratio (mol/mol), and C is enzyme concentration based on oil weight (wt.%).

The results obtained were than analyzed by ANOVA to assess the goodness of fit. The ANOVA of the model and respective model terms are tabulated in Table 3. The Model F-value of 31.80 implies the model is significant. There is only a 0.01% chance that a "Model F-Value" this large could occur due to noise, indicating that the quadratic model is reliable in predicting the FAME yield. Values of "Prob > F" less than 0.0500 indicate model terms are significant. In this study, it was observed that all the linear and quadratic terms of water content (A), molar ratio of methanol to oil (B), and enzyme dosage (C) had a significant effect on the FAME yield. Besides that, the effect of interaction between water content and molar ratio of methanol to oil (AB) also affected the FAME yield significantly. To test the fit of the model, the coefficient of determination (*R*^*2*^) were evaluated, which was 0.9662 indicating that the model does not explain only 3.38% of total variations. The adjusted determination coefficient (Adj *R*^*2*^ = 0.9359) was also high to advocate for a high significance of the model.

**Table 3:** ANOVA of the model and respective model terms

**Source**	**Degree of freedom**	**Mean square**	**F-Value**	**Prob > F**	**Remarks**
Model	9	336.95	31.80	< 0.0001	Significant
A	1	1193.46	112.63	< 0.0001	Significant
B	1	233.52	22.04	0.0008	Significant
C	1	106.21	10.02	0.0101	Significant
AB	1	55.13	5.20	0.0457	Significant
AC	1	44.18	4.17	0.0684	Not significant
BC	1	3.12	0.29	0.5990	Not significant
A^2^	1	1068.61	100.85	< 0.0001	Significant
B^2^	1	422.01	39.83	< 0.0001	Significant
C^2^	1	97.47	9.20	0.0126	Significant
Residual	10	10.60			
Lack of fit	5	16.29	3.32	0.1068	Not significant
Pure error	5	4.90			

*R*^*2*^ = 0.9662, *R*^*2*^ (adjusted) = 0.9359.

**Figure 5:**
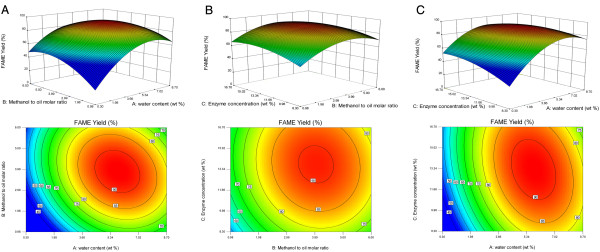
**(A-C) Response surface curves and contour plots of FAME yields showing the interactions among the initial water added based on oil weight (wt.%, A), methanol to tung oil molar ratio (mol/mol, B) and enzyme concentration based on oil weight (wt.%, C).** (**A**) Fixed level: C 12.5 wt. %; (**B**) Fixed level: A 4.5 wt.%; (**C**) Fixed level: B 3.5.

The entire relationships between reaction factors and response could be better understood by examining contour plots and response surface curves generated from the predicted model Eq. (1). As shown in Figure 5, all three contour plots were convex nature indicating that there were well-defined optimum operating conditions for the transesterification of FAME.

Usually, in nonaqueous media an optimum amount of water was needed for biocatalysis. Lipases possess the unique feature of acting at the aqueous and nonaqueous interface. Activation of lipase involves the opening of the lid on the oil–water interface, hence allowing substrates access to the active site [37]. Therefore, the transesterification yields and the activity of the lipase in nonaqueous media depend on the size of interfacial area which can be increased by the addition of certain amounts of water. However, since lipases usually catalyze hydrolysis in aqueous media, excess water may also stimulate the competing hydrolysis reaction. The optimum water content is a compromise between minimizing hydrolysis and maximizing enzyme activity for the transesterification reaction [38]. Figure 5A shows contour plots regarding the effects of water content, methanol to oil molar ratio and their interactions on FAME yield at a constant enzyme dosage of 12.5 wt.%. The elliptical curves of the contour plots indicated that the interaction between water and methanol was strong [39], where both water content and methanol content are critical parameters for lipase activity, and water affects the distribution of water molecules present in the reaction system. When the water content was in the range from 4.40 wt.% to 7.09 wt.% with the methanol to oil molar ratio between 2.87 and 4.92, the FAME yield would exceed 90%. With the water content as low as 0.3 wt.%, the lipase activity was very low and the FAME yield could only reach about 40%. With the increased addition of water there was a considerable increase in the ester production showing the enhancement in the activity of the enzyme. However, at the water content beyond 7.09 wt.%, the production of esters decreased. Tamalampudi *et al.*[40] also reported that the optimum water content was 5% (*v*/*v*) in *Jatropha* oil for producing FAME catalyzed by immobilized whole-cell *R. oryzae* lipase. The free *R. oryzae* lipase catalyzed the methanolysis of soybean oil in the presence of 4~30 wt.% water, while the lipase was nearly inactive in the absence of water [13]. In contrast *Candida antarctica* lipase (Novozym 435), which shows no interfacial activation without a lid covering the entrance to the active site [41], displayed the highest activity without addition of water [40].

Methanol concentration is another crucial parameter for biosynthesis of FAME. The Excess methanol would accelerate reaction rates, while on the other hand the poor solubility of methanol in oils and good solubility in water may lead to the loss of enzyme activity. Many researchers successfully improved the biodiesel yield by adding alcohol in a stepwise manner in solvent-free system to minimize inhibition of the enzyme [42]. In this study, experiments were performed at molar ratios of methanol to oil ranging from 0.98:1 to 6.02:1 by four-stepwise addition of methanol (Figure 5A and B). As was expected, an increase in the number of moles of methanol with respect to the oil resulted in an increase in the FAME yield. Ultimately, the formation of methyl esters reached a maximum level and further increases in the methanol to tung oil molar ratio resulted in a decrease in the formation of esters. This tendency was consistent with other researches which revealed that the optimum molar ratio of methanol to oil was 5:1 using immobilized *R. oryzae* lipase [14,36].

Enzyme dosage may also influence the conversion of the methyl esters. The contour plots and the response surface curves in Figure 5B and C show the predicted response surface of FAME yield as a function of enzyme dosage and water content or methanol to oil molar ratio. Enzyme loading in the range from 8.30 wt.% to 16.70 wt.% was examined in the transesterification of tung oil with methanol. The FAME yield above 90% was obtained in the presence of 10.83 wt.% to 15.70 wt.% enzyme at the water content between 4.40 wt.% and 7.09 wt.% with a fixed methanol to oil molar ratio of 3.5. Lower amount of immobilized enzyme would lead to lower FAME yields, while too much catalyst in the reaction system might cause mass transfer limitations. This behavior also has been observed by other researchers [40,43].

### Optimization analysis

The optimum conditions for the three variables were obtained using numerical optimization feature of Design-Expert 8.0.7.1. The software searches for a combination of factors that simultaneously satisfy the requirements placed on each of the response and factors. The goal was set to maximize the FAME yield in the high and low limit ranges of the three variables as stated in Table 1. The optimized condition calculated was 5.66 wt.% of the initial water added based on oil weight, 3.88 of methanol to tung oil molar ratio and 13.24 wt.% of enzyme concentration based on oil weight, under which the predicted FAME yield reached 92.6%. In order to verify the prediction of the model, the transesterification of tung oil with methanol using the immobilized chimeric r27AROL was carried out under the predicted optimal reaction condition. The experimental FAME yield was 91.9±2.5%, a figure well within the estimated value of the model equation. The results achieved here also confirmed the validity of the predicted model.

### Operational stability of the immobilized r27AROL

**Figure 6:**
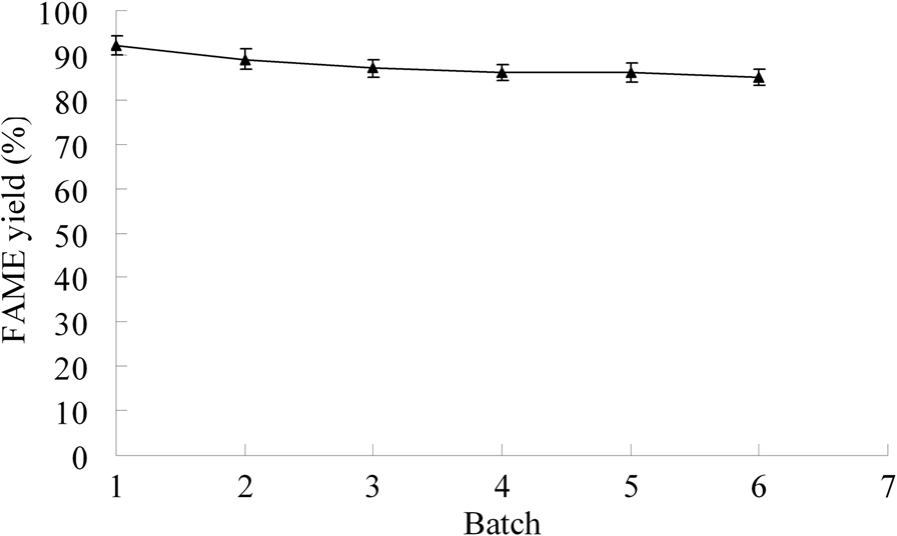
Operational stability of the immobilized r27AROL.

To further examine the potential of the immobilized r27AROL for FAME production, reusability of the lipase was investigated. After each batch reaction, the immobilized lipase was recovered by filtration and the next batch was carried out with fresh substrate. As shown in Figure 6, 85.1±1.8% of its original activity was maintained after being reused for six batches, which indicated a promising application for biodiesel production.

## Conclusion

Using tung oil as non-edible raw material and a chimeric lipase from *R. oryzae* as an economic catalyst make this study a promising one for biodiesel applications. In the present work, a chimeric lipase from *R. oryzae* was constructed by replacing the prosequence with that from *R. chinensis* lipase and successfully expressed in *P. pastoris* with the highest activity of 4050 U/mL, which was 11 fold higher than that of the parent. This is the first report on improving the expression level of the lipase from *R. oryzae* by replacing prosequences. The immobilized chimera was used successfully for biodiesel production from tung oil, which achieved higher FAME yield compared with the free chimeric lipase, non-chimeric lipase and mature lipase. By response surface methodology, three variables, water content, methanol to tung oil molar ratio and enzyme dosage were proved to be crucial parameters for biosynthesis of FAME and the FAME yield reached 91.9±2.5% at the optimized conditions by adding 5.66 wt.% of the initial water based on oil weight, 3.88 of methanol to tung oil molar ratio and 13.24 wt.% of enzyme concentration based on oil weight at 40°C.

## Methods

### Strains, plasmids and materials

*R. oryzae* XY1 suitable for biodiesel production was isolated from soil and conserved in our lab, which was used for ROL gene amplification. *P. pastoris* GS115 and plasmid pPIC9K were purchased from the Invitrogen Company. Tung oil was purchased from Luzhou Co. Ltd. (Sichuan, China) with acid value of 2.19 mg KOH/g, composed of 2.8% palmitic acid (C16:0), 1.5% of stearic acid (C18:0), 5.8% oleic acid (C18:1), 11.1% linoleic acid (C18:2), 1.7% linolenic acid (C18:3), 66.4% elaeostearic acid (C18:3), 1.6% eicosenoic acid (C20:1) and 9.2% behenic acid (C22:0). Methyl palmitate, methyl stearate, methyl oleate, methyl linoleate, methyl linolenate, methyl elaeostearate, methyl eicosenoate, methyl behenate and methyl heptadecanoate were purchased from Sigma Co. Ltd.. 9(Z),11(E),13(E)-Octadecatrienoic acid methyl ester (methyl elaeostearate) was purchased from Cayman Chemical Co. (USA). Anion exchange resin Amberlite IRA-93 was purchased from Cangzhou Co. Ltd. (Hebei, China). All other chemicals are of analytical grade.

### Cloning of *R. oryzae* lipase gene, construction of the lipase expression vectors and transformation

The open reading form of the *rol* gene was amplified directly from *R. oryzae* XY1 genomic DNA using a pair of primers, ROL-F1 (CCG**ATG**GTTTCATTCATTTCCA) and ROLR1 (GC**TTA**CAAACAGCTTCCTTCG) according to the sequence of the lipase from *R. oryzae* (GenBank accession no. AF229435). The PCR fragment was DNA sequenced, based on which the lipase gene without its own signal peptide (*proROL*) was amplified using a pair of primers, ROL-F2 (ATC*CCTAGG*GTTCCTGTTTCTGGTAAATC) and ROLR2 (CAGT*GCGGCCGC***TTA**CAAACAGCTTCCTTCG). The restriction sites *Avr*II and *Not*I were incorporated into the forward and reverse primer sequence, respectively. The PCR fragment was ligated into the respective sites of pPIC9K resulting in pPIC9K-proROL under the control of the methanol inducible alcohol oxidase 1 promoter (*P*_*AOX1*_) and fused in-frame with the α-factor secretion signal peptide of *S. cerevisiae*. For construction of the chimeric lipase gene, primers RCL-F2 (ATC*CCTAGG*GTTCCTGTTGCTGGTCATAAAGGTTC) and RCL-PROR (GCTGGGGGCAGTGGACGTAGCAGGAATAGG) were used to amplify the prosequence from *R. chinensis* lipase with pPIC9K-proRCL [28] as template; primers ROL-MatureF (TCCTGCTACGTCCACTGCCCCCAGCTCTGATGGTGGTAAGG) and ROLR2 were used to amplify the mature lipase gene from *R. oryzae* with pPIC9K-proROL as template; then these two PCR fragments were ligated by overlap extension polymerase chain reaction (OE-PCR) to generate the chimeric lipase gene *proAROL*. For expression of mROL, the mature lipase gene from *R. oryzae* was amplified using a pair of primers, ROL-mF (ATT*CCTAGG*TCTGATGGTGGTAAGGT) and ROLR2. The digested PCR fragments of *proAROL* and *mROL* by *Avr*II and *Not*I were ligated into the respective sites of pPIC9K resulting in pPIC9K-proAROL and pPIC9K-mROL, respectively. *P. pastoris* GS115 was transformed with *Bgl*II-linearized plasmids by electroporation, and selection of *His*^*+*^ transformants and geneticin resistant transformants was done on minimal dextrose medium (MD, Invitrogen BV) and YPD-G418 medium (Invitrogen BV), respectively. The positive transformants were confirmed by PCR.

### Expression of lipases in *P. pastoris*

Fermentation experiments were performed in a 7-L bioreactor (New Brunswick, BioFlo 110, USA) as described by Wu [29]. Briefly, in the glycerol batch phase, 200 mL of inoculum was directly added into 2.6 L of a Fermentation Basal Salts Medium and the fermentation conditions were maintained at 28°C, pH 5.0 and dissolved oxygen (DO) between 20 and 60% saturation. Until all of the glycerol was consumed, then the process was converted to a glycerol fed-batch phase, with feeding of 50% (*v/v*) glycerol containing 1.2% (*v/v*) trace solution at the average rate of 12.4 g/L/h. After the desired biomasses were reached, the methanol fed-batch phase was initiated, during which the culture was supplied with 100% (*v/v*) methanol containing 1.2% (*v/v*) trace solution, and the methanol concentration was controlled at 1 g/L by an on-line methanol analyzer (FC2002, Shanghai Super-xinxi, China).

### Lipase activity determination

Lipase activity was measured on emulsified *p*NPP according to Kordel *et al.*[44]. One enzyme unit was defined as the amount of enzyme releasing 1μmol of *p*-nitrophenol per minute under the assay conditions. And the lipase activity was measured by pH-stat assay using olive oil emulsion as substrate [18]. One unit was defined as the amount of enzyme required to release 1 mmol of fatty acid per minute under assay conditions. All the assays were done in triplicate, and significant differences (p*<*0.05) were measured.

### Lipase purification

The recombinant enzymes from the culture supernatant after cultivation for 76 h were purified through ultrafiltration, SP-Sepharose column, Phenyl-sepharose 6 FF column as described by Yu [28].

### Effect of temperature and pH on enzyme stability and activity

Optimal pH was determined by examining the activity of the enzyme at 40°C in the range of pH 5.0~11.0. The pH stability was measured by incubating lipase solution in buffers (50mM, pH 5.0~11.0) for 1 h at 25°C and analyzing the residual activity. Optimal temperature was determined by measuring the enzyme activity at optimal pH under various temperatures (20~60°C). Thermostability was determined by pre-incubating the purified enzyme in the temperature range of 20~60°C for 0.5 h. Lipase activity was determined by *p*NPP assay.

### Kinetic parameters

The interfacial kinetic parameters k*_cat_ and K*_m_ were determined in a heterogeneous medium using *p*NPP as substrate according to the method described by Burdette *et al.*[45]*.*

### Preparation of immobilized lipase

The crude lipase from supernatant of the culture was lyophilized and redissolved in 50mM Tris–HCl buffer (pH 8.5). The method for immobilization of the lipase was described by Wang *et al.*[14]. Briefly, the macroporous anion exchange resin Amberlite IRA-93 (1 g) and the lipase (2 mg) were mixed together in 50mM Tris–HCl buffer (pH 8.5) at 20°C for 4 h at 160 rpm. These particles above were filtered, and then mixed with fresh lipase (2 mg) and glutaraldehyde (0.5 (*v/v*) % based on total volume) in 5 mL buffer at 28°C for 20 min. The immobilized lipase was rinsed thrice with 15mL Tris–HCl buffer [2]. The activity of the immobilized lipase (U/g support) was 65 U/g.

### Enzymatic methanolysis reaction

The methanolysis reaction carried out with the immobilized lipase or the free lipases in the solvent-free system in screw-capped 50-mL vials on a shaker at 160 rpm and 40°C for 48 h, unless indicated specifically. The reaction mixture contained 10 g tung oil, a certain amount of methanol, enzyme and water with a total volume around 13 mL. Details about molar ratio of methanol to oil, enzyme concentration based on oil weight (wt.%), and the initial water added based on oil weight (wt.%) were specified for each case. Methanol was added by four steps. The first portion of methanol and 10 g of oil were added at the start of the reaction; the second, the third and the fourth portion of methanol were added at an interval of 8 h. Reactions were performed in triplicate.

### Gas chromatography analysis

The FAME contents in the reaction mixture were quantified using a gas chromatography (Agilent 7890, USA) equipped with a flame ionization detector (FID). The column was a Nukol capillary column (0.25 mm × 60 m, Supelco, USA). Helium was used as a carrier gas. The column temperature was kept at 100°C for 1 min, heated to 230°C at 4°C /min, held at that degree for 25 min. The temperatures of the injector and detector were set at 300°C. Methyl heptadecanoate served as the internal standard for GC. Aliquots of 100 μL samples were taken from the reaction mixture and centrifuged at 12 000 rpm for 10 min to obtain the upper layer, and were diluted in n-hexane and mixed with methyl heptadecanoate. Then, analyses were done by injecting 1 μl sample into the column. Methyl palmitate (C16:0), methyl stearate (C18:0), methyl oleate (C18:1), methyl linoleate (C18:2), methyl linolenate (C18:3), methyl elaeostearate (C18:3), methyl eicosenoate (C20:1) and methyl behenate (C22:0) were used as standards. FAME yield (%) was defined as FAME amount produced divided by initial amount of oil (g/g).

### Central composite rotatable design and statistical analysis

RSM was used to evaluate the best conditions for the FAME yield. A five-level-three-factor CCRD, including six replicates at the center point, totaling 20 assays, was adopted to evaluate the effects of enzyme dosage, molar ratio of methanol to oil and water content on the FAME yield of the reaction in this study. The corresponding CCRD was shown in Table 1. The data obtained were fitted to a second-order polynomial equation using the software Design-Expert 8.0.7.1:

(2) $$Y=\beta_{0}+\sum_{i=1}^{3}\beta_{i}X_{i}+\sum_{i=1}^{3}\beta_{\mathrm{ii}}X_{i}^{2}+\sum_{i=1}^{2}\sum_{j=i+1}^{3}\beta_{\mathrm{ij}}X_{i}X_{j}$$

where *Y* is the response (% FAME yield), *β*_*0*_, *β*_*i*_, *β*_*ii*_ and *β*_*ij*_ are the regression coefficients for the intercept, linear, quadratic and interaction terms, respectively and *X*_i_ and *X*_j_ are the independent variables. Design-Expert 8.0.7.1 was used for analysis of variance (ANOVA) and predicting the optimal conditions for the enzymatic reaction.

### Operational stability of the immobilized r27AROL

Each batch of the reaction was carried out at the optimized conditions by adding 5.66 wt.% of the initial water based on oil weight, 3.88 of methanol to tung oil molar ratio and 13.24 wt.% of enzyme concentration based on oil weight at 40°C for 48 h. After each batch, the immobilized r27AROL was recovered by filtration, washed two times with cold hexane, then vacuum-dried before use in the subsequent batch.

## Abbreviations

ROL: The *R. oryzae* lipase; proROL: The *R. oryzae* prolipase; proAROL: The chimeric *R. oryzae* prolipase; r27AROL: The truncated chimeric *R. oryzae* prolipase secreted by *Pichia pastoris*; r28ROL: The truncated non-chimeric *R. oryzae* prolipase secreted by *Pichia pastoris*; mROL: The mature *R. oryzae* lipase; RCL: The *R. chinensis* lipase; FAME: Fatty acid methyl esters; RSM: Response surface methodology; CCRD: Central composite rotatable design; DCW: Dry cell weight; FID: Flame ionization detector; pNPP: *p-*nitrophenyl palmitate

## Competing interests

The authors declare that they have no competing interests.

## Authors’ contributions

YXW and XY designed the research and prepared the manuscript. YXW, SC, GYL did the experiments. RX helped to revise the manuscript. All authors read and approved the final manuscript.
